# Airway Eosinophilia on Bronchoalveolar Lavage and the Risk of Exacerbations in COPD

**DOI:** 10.3390/biomedicines10061412

**Published:** 2022-06-15

**Authors:** Chunman Germain Ho, Stephen Milne, Xuan Li, Chen Xi Yang, Fernando Sergio Leitao Filho, Chung Yan Cheung, Julia Shun Wei Yang, Ana I Hernández Cordero, Cheng Wei Tony Yang, Tawimas Shaipanich, Stephan F van Eeden, Janice M Leung, Stephen Lam, Don D Sin

**Affiliations:** 1 Centre for Heart Lung Innovation, St Paul’s Hospital, The University of British Columbia, Vancouver, BC V6Z 1Y6, Canada; germainh@student.ubc.ca (C.G.H.); annie.li@hli.ubc.ca (X.L.); yolanda.yang@hli.ubc.ca (C.X.Y.); fernandosergioepm@gmail.com (F.S.L.F.); chung.cheung@hli.ubc.ca (C.Y.C.); julia.yang@hli.ubc.ca (J.S.W.Y.); ana.hernandez@hli.ubc.ca (A.I.H.C.); tony.yang.phd@gmail.com (C.W.T.Y.); janice.leung@hli.ubc.ca (J.M.L.); don.sin@hli.ubc.ca (D.D.S.); 2 Division of Respiratory Medicine, The University of British Columbia, Vancouver, BC V6T 1Z3, Canada; tshaipanich@providencehealth.bc.ca (T.S.); stephan.vaneeden@hli.ubc.ca (S.F.v.E.); slam@bccancer.bc.ca (S.L.); 3 Faculty of Medicine and Health, The University of Sydney, Camperdown, NSW 2006, Australia; 4 Providence Airway Centre, St Paul’s Hospital, Vancouver, BC V6Z 1Y6, Canada; 5 BC Cancer Research Centre, Department of Integrative Oncology, Vancouver, BC V5Z 1L3, Canada

**Keywords:** biomarkers, chronic obstructive pulmonary disease, eosinophilia, bronchoscopy

## Abstract

The associations between airway eosinophilia, measured in sputum or peripheral blood, and acute exacerbations of chronic obstructive pulmonary disease (AECOPD) are inconsistent. We therefore aimed to determine the association between eosinophilia in bronchoalveolar lavage (BAL) fluid and AECOPD in a clinical cohort. We analyzed differential cell counts from baseline BAL fluid in participants in the DISARM clinical trial (Clinicaltrials.gov #NCT02833480) and classified participants by the presence or absence of BAL eosinophilia (>1% of total leukocytes). We determined the association between BAL eosinophilia and AECOPD over 1 year of follow-up using negative binomial regression and Cox proportional hazards test. N = 63 participants were randomized, and N = 57 had BAL differential cell counts available. Participants with BAL eosinophilia (N = 21) had a significantly increased rate of acute exacerbations (unadjusted incidence rate ratio (IRR) 2.0, *p* = 0.048; adjusted IRR 2.24, *p* = 0.04) and a trend toward greater probability of acute exacerbation (unadjusted hazard ratio (HR) 1.74, *p* = 0.13; adjusted HR 2.3, *p* = 0.1) in the year of follow-up compared to participants without BAL eosinophilia (N = 36). These associations were not observed for BAL neutrophilia (N = 41 participants), BAL lymphocytosis (N = 27 participants) or peripheral blood eosinophilia at various threshold definitions (2%, N = 37; 3%, N = 27; 4%, N = 16). BAL may therefore be a sensitive marker of eosinophilic inflammation in the distal lung and may be of benefit for risk stratification or biomarker-guided therapy in COPD.

## 1. Introduction

Chronic obstructive pulmonary disease (COPD) is characterized by irreversible airflow obstruction and acute exacerbations (AECOPD). Repeated AECOPD events can worsen lung function decline [1,2] and impact morbidity and mortality [3,4]. Identifying patients at increased risk of AECOPD and initiating effective preventative treatments is therefore a fundamental management goal [5].

Eosinophilic inflammation is present in up to 40% of patients with COPD [6]. Sputum and blood eosinophilia are associated with an increased risk of AECOPD [7,8], but the findings are inconsistent. This may be because neither sputum nor blood samples are representative of the distal lung microenvironment, which is the predominant site of disease in COPD [9]. We therefore determined the association between distal airway eosinophilia measured by bronchoalveolar lavage (BAL) and the risk of AECOPD in a prospective COPD cohort.

## 2. Methods

*DISARM trial:* We analyzed data from participants in the DISARM study (Clinicaltrials.gov #NCT02833480), which investigated the effects of a 12-week treatment with combined inhaled corticosteroid/long-acting beta-2-agonist (ICS/LABA) vs. LABA alone on the lung microbiome in participants with stable COPD [10]. A description of the DISARM trial is provided in the Detailed Methods section (Supplementary File S1), and gene sequencing data are available in the National Center for Biotechnology Information’s Sequence Read Archive (PRJNA685554) [11] and Gene Expression Omnibus (GSE162120) [12] repositories. Peripheral blood was collected from participants at baseline, and BAL fluid (via bronchoscopy) was collected following a four-week run in with LABA monotherapy. Randomization occurred one week following the bronchoscopy. Following completion of the trial, each participant was followed prospectively to record AECOPD data and vital status for a minimum of one year from the date of enrollment. The study was approved by the University of British Columbia/Providence Health Care Human Research Ethics committee (approval #H14-02277).

*Bronchoscopy procedures:* The details of our standardized research bronchoscopy protocol have been published previously [13]. After providing informed consent and under light sedation, a fiberoptic bronchoscope (Olympus Corporation, Tokyo, Japan) was passed into the airways via the mouth. BAL was performed preferentially on the right middle lobe or lingula, by instilling aliquots of warm sterile saline until a return volume of 30 mL or total instilled volume of 200 mL was reached.

*BAL processing:* Whole BAL fluid was strained and centrifuged, and the cell pellet resuspended in Dulbecco’s phosphate-buffered saline. Slides containing 50,000 cells were stained with modified Wright-Giemsa stain. Differential cell counts were performed by two independent, trained observers. We defined BAL eosinophilia and neutrophilia as eosinophils and neutrophils >1% of total BAL leukocyte count, respectively [14]. Due to the distribution of lymphocyte counts in our data, we defined lymphocytosis as greater than the median lymphocyte count (>2.25% of total BAL leukocyte count).

*Peripheral blood processing:* Peripheral blood was collected by venipuncture, and automated cell counts were performed using the ADVIA 2120i Hematology System (Siemens Healthcare Diagnostics, Tarrytown, NY, USA). We defined peripheral blood eosinophilia as >3% of total blood leukocyte count [15].

*Statistical analysis:* For the primary analysis, we determined the relationship between eosinophilia and AECOPD events in the follow-up year (excluding the first month where bronchoscopy-induced AECOPDs were possible) using negative binomial regression. This model was chosen due to over-dispersion of the outcome variable. We used stepwise selection of covariates from an a priori list of potential confounders, including age, sex, body mass index (BMI), smoking status, and treatment group allocation. Forced expiratory volume in 1 s (FEV_1_), which is a known predictor of exacerbation risk, was specifically excluded due to its correlation with BAL eosinophil count, so as not to introduce multicollinearity. We quantified the effects by the incidence rate ratio (IRR) (i.e., the exponent of the model estimate) and model fit using the Akaike information criterion (AIC). In secondary analyses, we examined the effects of eosinophilia on AECOPD events over time using the Kaplan–Meier curves. We used Cox proportional hazards models to determine the probability of the first AECOPD event, with and without adjustment for the covariates listed above, expressed as the hazard ratio (HR). We repeated each of the analyses for BAL neutrophilia and lymphocytosis. Finally, we compared BAL and blood eosinophil proportions using Spearman’s correlation. All analyses were performed using R (version 4.0.5, R Foundation for Statistical Computing, Vienna, Austria). Statistical significance was set at *p* < 0.05.

## 3. Results

Sixty-three participants underwent bronchoscopy and randomization. We performed differential cell counts on BAL fluid from 57 participants for whom adequate lavage fluid volume was available (6 participants returned less than 10 mL BAL fluid). The majority of participants were males (84.2%) with a median (range) age of 64 (48–82) years and moderate airflow obstruction (mean post-bronchodilator FEV_1_ of 59.8 (18.1)% predicted). Almost half were current smokers. Using a threshold of >1% of total leukocytes [14], 21 participants had BAL eosinophilia, and 36 participants had no BAL eosinophilia; the groups had similar characteristics, including respiratory medication use, at baseline (Table 1).

**Figure 1:**
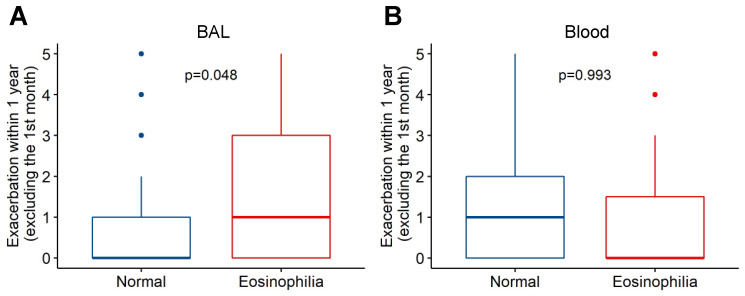
Relationship between eosinophilia and AECOPD events in the DISARM trial. BAL eosinophilia (>1% of total BAL leukocytes) (**A**), but not peripheral blood eosinophilia (>3% of total blood leukocytes) (**B**), was associated with a higher rate of AECOPD events (incidence rate ratio 2.0, *p* = 0.048 vs. 1.0, *p* = 0.99). Abbreviations: AECOPD, acute exacerbations of chronic obstructive pulmonary disease; BAL, bronchoalveolar lavage.

**Figure 2:**
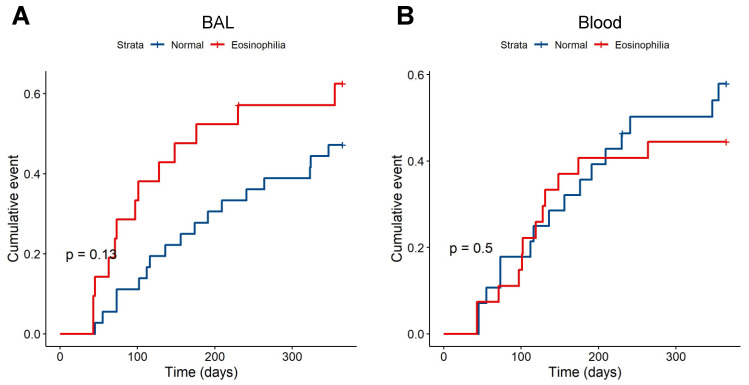
Survival analysis of the effect of eosinophilia on AECOPD events in the DISARM trial. BAL eosinophilia (**A**), but not peripheral blood eosinophilia (**B**), was associated with a non-significant increased probability of an AECOPD event (Cox proportional hazards test unadjusted hazard ratio 1.74, *p* = 0.13 vs. 0.77, *p* = 0.5). Abbreviations: AECOPD, acute exacerbations of chronic obstructive pulmonary disease; BAL, bronchoalveolar lavage.

We determined the association between BAL eosinophilia and subsequent AECOPD events using negative binomial regression. BAL eosinophilia was associated with a higher rate of AECOPD events in the year of follow-up (median 0 vs. 1; IRR = 2.0, *p* = 0.048, model AIC = 171) (Figure 1A). This remained significant in the fully adjusted model that included age, sex, BMI, smoking status, and treatment allocation as covariates (IRR = 2.24, *p* = 0.04, model AIC = 181). We also determined the effect of BAL eosinophilia on the probability of an AECOPD event during follow-up using Cox proportional hazards models. The unadjusted model showed a non-significant increase in the risk of AECOPD with BAL eosinophilia over time (unadjusted HR = 1.74, *p* = 0.13). When fully adjusted, a non-significant increase in the risk of AECOPD remained (adjusted HR = 2.3, *p* = 0.1) (Figure 2A). There were no associations with AECOPD for BAL neutrophilia (>1% total leukocytes: unadjusted IRR = 1.42, *p* = 0.40, AIC = 174; unadjusted HR = 1.09, *p* = 0.83) or lymphocytosis (> median lymphocyte proportion: unadjusted IRR = 0.79, *p* = 0.51, AIC = 174; unadjusted HR = 1.0, *p* = 0.99).

**Figure 3:**
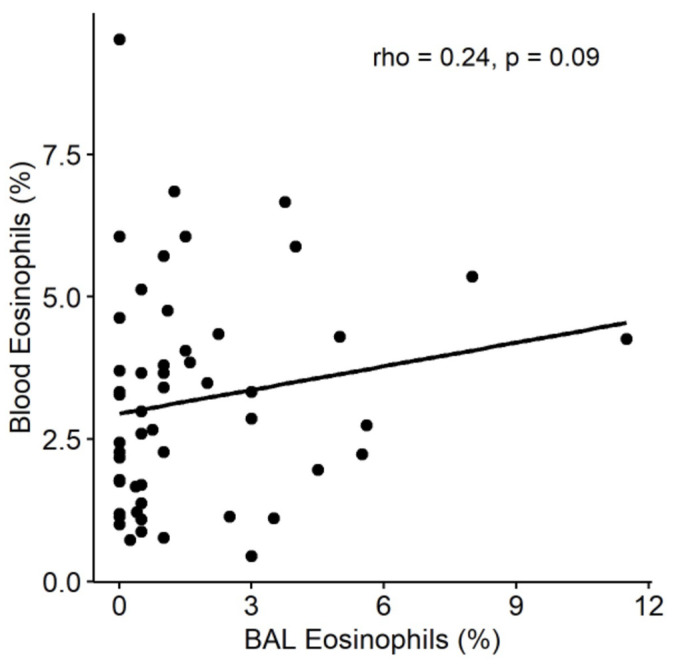
Relationship between BAL and peripheral blood eosinophil counts at baseline in the DISARM trial. There was no correlation between eosinophil proportions in the two compartments (Spearman’s rho 0.24, *p* = 0.09). Abbreviations: BAL, bronchoalveolar lavage.

We used a similar approach to investigate the association between peripheral blood eosinophilia (defined as >3% of total leukocytes) on AECOPD during follow-up. Notably, there was no association between peripheral blood eosinophilia and the rate of AECOPD events (unadjusted negative binomial model, IRR = 1.0, *p* = 0.99) (Figure 1B) or probability of exacerbation (unadjusted Cox proportional hazards model, HR = 0.77, *p* = 0.5) (Figure 2B). Because there is no clear consensus on the most appropriate threshold to define peripheral blood eosinophilia in COPD [15,16], we conducted sensitivity analyses by using thresholds of >2% and >4% eosinophils, both of which produced similar results (2% threshold: IRR = 1.08, *p* = 0.86 and HR = 0.87, *p* = 0.73; 4% threshold: IRR = 1.1, *p* = 0.81 and HR = 0.86, *p* = 0.72). Finally, in order to explore the discordant associations of BAL and peripheral blood eosinophilia with AECOPD, we examined the relationship between these two cell counts. There was no significant correlation between BAL and blood eosinophil proportions (Spearman’s rho = 0.24, *p* = 0.09) (Figure 3).

## 4. Discussion

Our analysis showed that airway eosinophilia, but not peripheral blood eosinophilia, was associated with an increased risk of AECOPD in a cohort of stable COPD patients. Peripheral blood eosinophilia has been associated with increased exacerbation risk in some studies [7], while others have found no such association [17]. These inconsistencies may potentially be explained by the lack of correlation between eosinophil proportions in blood and BAL fluid demonstrated here, which suggests that peripheral blood eosinophilia is a weak surrogate marker of eosinophilic airway inflammation. Another possible explanation is that previous studies have used inconsistent threshold values for defining blood eosinophilia [15,16], and there is currently no consensus on the optimal threshold value. We used sensitivity analyses to examine different blood eosinophil thresholds but found no association with exacerbation risk even at the highest threshold value of >4%. Additionally, previous studies may have been confounded by corticosteroid use among participants. In our study, all participants were free of systemic and inhaled corticosteroids for at least 1 month prior to bronchoscopy, meaning the BAL counts were unlikely to have been influenced by the effects of steroids.

Stratification of patients according to future exacerbation risk is an integral part of COPD management [5]. Even though a prior history of exacerbation remains the most reliable prognosticator of future exacerbation risk [17], objectively measured biomarkers of exacerbation risk may be used to further stratify patients in both clinical and research settings. Additionally, biomarkers of exacerbation risk may be of benefit for targeted therapy with the aim of reducing the number needed to treat and limiting the potential unwarranted side effects. To our knowledge, no previous studies have examined exacerbation risk in the context of BAL eosinophilia.

Unlike other biomarkers of AECOPD risk, such as reduced FEV_1_ [17] or increased plasma fibrinogen [18], the link between airway eosinophilia and AECOPD risk points toward a distinct immunopathological state in the distal lung that may be driving the risk. This is further supported by the lack of association with neutrophilia and lymphocytosis in BAL. However, the precise mechanisms underlying this association are still unclear. Sputum eosinophilia has been associated with clinical response to corticosteroids in stable COPD [19,20]; our finding that eosinophilic inflammation measured directly from the airway by BAL is associated with future AECOPD risk may therefore have important therapeutic implications. Interestingly, clinical trials of biologic therapies that specifically target eosinophils (e.g., anti-interleukin-5 monoclonal antibodies) have shown little effect in unselected COPD patients [21]. However, none of the previous studies have used BAL eosinophil counts to guide anti-eosinophilic therapies. Even though bronchoscopy is a relatively invasive sampling method, and non-invasive methods such as sputum, blood sampling, or lung function testing would be more clinically practical, we believe that the potential for BAL eosinophilia to guide such therapies warrants investigation in clinical trials.

A particular strength of our study was the absence of confounding effects of inhaled or systemic corticosteroid therapy for at least one month prior to bronchoscopy. These treatments may distort the relationship between eosinophil count and the rate of AECOPD, although it should be noted that their therapeutic efficacy and their effects on eosinophil measurements are both highly variable. We accounted for the use of ICS during the study period by including treatment allocation as a covariate in our models. We also collected exacerbation data prospectively and well beyond the completion of the clinical trial; all exacerbation events were recorded by specialist respiratory physicians in the participant’s electronic medical record. This ensured a robust exacerbation data set that is not confounded by long-term recall bias.

The main limitation of our data was the relatively small sample size, which likely underpowered the statistical models. The clinical trial on which this post hoc analysis is based was powered to detect a change in the microbiome following ICS/LABA treatment and was not powered for BAL cell content or exacerbations. However, the models for BAL eosinophilia produced consistent trends, which were not seen in the models for peripheral blood eosinophilia. Identifying these trends despite a small sample size suggests that BAL eosinophilia may be a sensitive marker of exacerbation risk, but this needs to be confirmed in a larger population. Additionally, peripheral blood and BAL were collected at different time points (four weeks apart). This may have contributed to the poor correlation between eosinophil counts from these two compartments but is unlikely to have influenced their relationships with AECOPD over the following year. The small sample size also did not allow sub-analysis by, for example, pre-enrollment respiratory treatment or exacerbation severity. Another limitation was the lack of accurate information on medication use during the follow-up period. It is not known whether participants in each of the BAL subgroups maintained their assigned medications beyond the 12-week trial, and it is likely that ongoing use of ICS-containing medications would have modified the rate of AECOPD in some patients. Finally, we used an arbitrary threshold (>1%) for defining BAL eosinophilia, which was extrapolated from measurements performed in healthy non-smokers [14]. The range of normal for eosinophils in BAL may be disease specific, and the optimum threshold for predicting exacerbation risk in COPD needs to be determined in a larger sample size.

## 5. Conclusions

These novel findings suggest that BAL eosinophilia is a sensitive marker of future AECOPD risk. If this is confirmed, measuring BAL eosinophils may be a valuable means of risk stratifying people with COPD and may ultimately facilitate a precision medicine approach to managing this disease.

## Figures and Tables

**Table 1:** Characteristics of COPD participants according to presence of BAL eosinophilia. All data are presented as mean (standard deviation) or percentage of column totals, unless otherwise specified. ^†^ Six and ^‡^ eight out of sixty-three randomized participants did not have differential cell counts available from BAL and peripheral blood, respectively. Abbreviations: COPD, chronic obstructive pulmonary disease; BMI, body mass index; LAMA, long-acting muscarinic antagonists; ICS, inhaled corticosteroids; BD, bronchodilator; FEV_1_, forced expiratory volume in 1 s; SGRQ, St George’s Respiratory Questionnaire; AECOPD, acute exacerbations of COPD.

	Whole Group N = 57 ^†^	No BAL Eosinophilia (≤1%) N = 36	BAL Eosinophilia (>1%) N = 21
**Age, median (range) years**	64 (48–82)	64 (48–80)	66 (52–82)
**Male, %**	84.2	83.3	85.7
**BMI, kg/m^2^**	25.6 (5.7)	26.3 (5.9)	24.3 (5.2)
**Current smokers, %**	47.4	52.8	38.1
**Smoking exposure, pack years**	45.8 (20.9)	47.6 (22.8)	42.5 (16.8)
**Post-BD FEV_1_, % of predicted**	59.8 (18.1)	63.7 (18.0)	52.8 (16.5)
**GOLD class, n (%)**			
I	5 (9)	4 (11)	1 (5)
II	27 (47)	18 (50)	9 (43)
III	22 (39)	13 (36)	9 (43)
IV	3 (5)	1 (3)	2 (9)
**SGRQ, total score**	41.5 (17.1)	40.7 (17.8)	42.6 (16.3)
**Medications at enrollment, %**			
LAMA	84.2	80.6	90.5
ICS	57.9	55.6	61.9
**Pre-treatment inflammatory status,** **n abnormal/n normal**			
Peripheral blood eosinophilia (>2%) ^‡^	37/18		
Peripheral blood eosinophilia (>3%) ^‡^	27/28		
Peripheral blood eosinophilia (>4%) ^‡^	16/39		
BAL neutrophilia (>1%) ^†^	41/16		
BAL lymphocytosis (>2.25/μL) ^†^	27/30		
**Treatment allocation following randomization, n**			
Formoterol	20	9	11
Formoterol/budesonide	19	16	3
Salmeterol/fluticasone	18	11	7
**Number of AECOPD events in year of follow-up, median (range)**	1 (0–5)	0 (0–5)	1 (0–5)

## Data Availability

Data from the DISARM trial are available on the National Center for Biotechnology Information’s Sequence Read Archive (SRA) under the BioProject number PRJNA685554 [11] (microbiome data) and the Gene Expression Omnibus (GEO) under accession number GSE162120 [12]. Other data are available from the investigators upon reasonable request.

## Supplementary Materials

The following supporting information can be downloaded at: https://www.mdpi.com/article/10.3390/biomedicines10061412/s1, Supplementary File S1: “Detailed Methods” (Supplementary_File_S1_Detailed_Methods.docx).
