# Supplementary material for: Airway Eosinophilia on Bronchoalveolar Lavage and the Risk of Exacerbations in COPD
Source: Biomedicines. 2022 Jun 15;10(6):1412. doi: 10.3390/biomedicines10061412 (PMC9220207; doi:10.3390/biomedicines10061412)
Supplement: Supplementary file 1 [file biomedicines-10-01412-s001.zip › biomedicines-1776783-supplementary.pdf]

SUPPLEMENTARY FILE S1: Detailed Methods

Airway eosinophilia on bronchoalveolar lavage and the risk of exacerbations in COPD

Chun-man Germain Ho BSc[1], Stephen Milne MBBS PhD[1,2,3], Xuan Li MSc[1], Chen Xi Yang MSc[1], Fernando Sergio Leitao Filho MD, PhD[1,2], Chung Yan Cheung PhD[1,4], Julia Shun-Wei Yang MSc[1], Ana I Hernández Cordero PhD[1], Cheng Wei Tony Yang PhD [1,4], Tawimas Shaipanich MD[2], Stephan F van Eeden MD, PhD[1,2], Janice M Leung MD[1,2], Stephen Lam MD[2,5], Don D Sin MD[1,2]

1. The University of British Columbia Centre for Heart Lung Innovation, St Paul's Hospital, Vancouver, British Columbia, Canada
2. The University of British Columbia Division of Respiratory Medicine, Vancouver, British Columbia, Canada
3. The University of Sydney Faculty of Medicine and Health, Sydney, New South Wales, Australia
4. Providence Airway Centre, Vancouver, British Columbia, Canada
5. BC Cancer Research Centre Department of Integrative Oncology, Vancouver, British Columbia, Canada

CORRESPONDING AUTHOR:
Dr. Stephen Milne
UBC Centre for Heart Lung Innovation
St. Paul's Hospital
1081 Burrard Street,
Vancouver V6Z 1Y6
British Columbia
Canada
E: Stephen.milne@hli.ubc.ca

Detailed Methods

The Study to Investigate the Differential Effects of Inhaled Symbicort and AdvaiR on Lung Microbiota (DISARM) trial: This randomized controlled trial was conducted to determine the effects of inhaled corticosteroid/long-acting beta-agonist (ICS/LABA) combination therapy on the lower airway microbiome in participants with COPD. The trial protocol was registered at clinicaltrials.gov (#NCT02833480) and the primary outcome results have recently been published.[1] Between October 2015 and June 2019, we recruited male and female participants

aged 40-85 years with COPD defined by a post-bronchodilator ratio of forced expiratory volume in 1 s to forced vital capacity (FEV1/FVC) <0.7, smoking history ≥10 pack years, and either FEV1 between 20% and 80% of predicted or clear evidence of emphysema on thoracic computed tomography. All participants were clinically stable without the use of systemic corticosteroids or antibiotics for at least 8 weeks prior to enrolment. Peripheral blood was collected at baseline. After ceasing any ICS therapy and undergoing a 4-week run-in period of LABA monotherapy (formoterol 12 microg via Turbuhaler twice daily), the purpose of which was to wash out any residual ICS, participants underwent bronchoscopy. One week after the bronchoscopy, participants were randomized 1:1:1 to receive formoterol 12 microg via Turbuhaler® twice daily, budesonide/formoterol 400/12 microg via Turbuhaler® twice daily, or fluticasone propionate/salmeterol 250/50 microg via Diskus® twice daily, for 12 weeks. At the end of the treatment period, participants returned for a second bronchoscopy. Neither the participants nor study coordinators were blinded to the treatment allocation, but study physicians assessing the participants during follow-up were unaware of their allocation. Acute exacerbations of COPD (AECOPD) were defined as acute worsening of symptoms requiring antibiotics and/or systemic corticosteroids. Data on AECOPD events and vital status were recorded prospectively after enrollment, for a minimum of one year. Data were confirmed from the electronic medical records following clinical review by specialist respiratory physicians.

Reference

1. Filho FSL, Takiguchi H, Akata K, Ra SW, Moon J-Y, Kim HK, et al. Effects of Inhaled Corticosteroid/Long-Acting β2-Agonist Combination on the Airway Microbiome of Patients with Chronic Obstructive Pulmonary Disease: A Randomized Controlled Clinical Trial (DISARM). Am J Respir Crit Care Med. 2021;204(10):1143-52.
